# Methodological and Measurement Advances in Social Determinants of HIV: View from NIH

**DOI:** 10.1007/s10461-021-03234-8

**Published:** 2021-03-29

**Authors:** Gregory Greenwood, Paul Gaist, Ann Namkung, Dianne Rausch

**Affiliations:** 1 Division of AIDS Research, National Institute of Mental Health, 5601 Fishers Lane, 9G19, Bethesda, MD 20852 USA; 2 Office of AIDS Research, National Institute of Health, Bethesda, MD 20852 USA; 3 Basic Sciences Program, National Institute of Allergy and Infectious Diseases, Bethesda, MD 20852 USA

**Keywords:** Social determinants, HIV

## Abstract

Social determinants are increasingly understood as key contributors to patterns of heightened risk for HIV acquisition and suboptimal care and treatment outcomes. Yet, the ability to rigorously model, map and measure these nuanced social dynamics has been a challenge, resulting in limited examples of effective interventions and resource allocation. In 2016, the United States National Institute of Mental Health (NIMH) and the National Institute of Allergy and Infectious Diseases (NIAID) issued a Request for Applications calling for methodological innovations around the social determinants of HIV. In May of 2019, NIMH, in collaboration with American University’s Center on Health, Risk and Society and the DC Center for AIDS Research, sponsored a symposium to bring together the funded teams to share accomplishments, distill lessons learned and reflect on the state of the science with other key stakeholders. Presentations focused on causal inference, multi-level analysis and mathematical modeling (Models); geospatial analytics and ecological momentary assessments (Maps); and measurement of social and structural determinants including inequalities and stigmas (Measures). Cross-cutting and higher-level themes were discussed and largely focused on the importance of critical and careful integration of social theory, community engagement and mixed methodologies into research on the social determinants of HIV.

## Introduction

Three decades into the HIV epidemic, the social conditions and economic circumstances that influence health, or social determinants, are increasingly understood as key contributors to patterns of heightened risk for HIV acquisition and suboptimal care and treatment outcomes. The social determinants of health have been defined by the WHO as, “the conditions in which people are born, grow, work, live, and age, and the wider set of forces and systems shaping the conditions of daily life [1] The HIV epidemic is a prime exemplar of the degree to which social and material circumstances determine vulnerability to illness and trends in access to treatment and sustained health. Across distinct geographic epidemic settings, morbidity and mortality statistics show that HIV continues to have its greatest impact among the poor, disenfranchised and stigmatized [2]. Curbing these inequities presents not only a societal imperative, but a centrally important scientific challenge. As noted by the recently updated National HIV AIDS Strategy for the United States [3] and the newly launched “Ending the HIV Epidemic: A Plan for America” initiative [4], stigma remains a primary challenge for those living with or at risk of HIV. They note that responding to HIV is not just a biomedical issue, but a social challenge as well. These conclusions echo those made by international agencies including the Joint United Nations Programme on HIV/AIDS (UNAIDS). The UNAIDS 2016–2021 strategy, which is tied to the United Nations Sustainable Development Goals, calls for reductions in social inequalities, including gender equality, and the elimination of HIV stigma and discrimination [5]. By understanding how these forces are produced and reproduced in dynamic every-day social interactions and engagement with larger institutional structures, the need and the possibilities for social change to address these constraints become illuminated.

Increased appreciation of the importance of social determinants has been matched by a recognition within the HIV field of the need for methodologies that can effectively assess the nuanced dynamics of the social determinants of HIV. Without such methodologies, development and testing of effective interventions remain limited. In 2016, the United States National Institute of Mental Health (NIMH) and the National Institute of Allergy and Infectious Diseases (NIAID), both institutes of the National Institutes of Health (NIH), issued a Request for Applications (RFA-MH16-200 and RFA-MH16-205) calling for methodological innovations to better understand and address the social determinants of HIV, including HIV prevention, care and treatment outcomes. Its goal was to further research in support of future program and policy responses to the epidemic across populations and settings [6, 7].

The salience of the research funded through this RFA and the methodological and conceptual challenges now being addressed through its collective portfolio has only increased in the last three years. Social and economic conditions play out in peoples’ daily lives shaping their potentially increased and disproportionate risk for HIV or worse health outcomes if they are living with the virus. Despite the availability of reliable, effective treatment for more than twenty years, less than half of those living with HIV worldwide are virally suppressed and in 2017 nearly a million individuals died from HIV [8]. Challenges in achieving viral suppression and improving treatment outcomes are linked to social determinants which underlie disparities in access to antiretroviral therapy (ART), viral suppression and HIV prognosis [9–13]. The reach of highly effective prevention is also suboptimal. HIV prevalence in certain geographic areas remains disproportionately high and among certain populations reaches over 70 percent [14]. Pre-exposure prophylaxis (PrEP) has yet to roll out in a majority of low resource international settings [15] and in the U.S., only approximately seven percent of high-risk individuals have been prescribed PrEP, with racial/ethnic minorities and women much less likely to receive a prescription [16]. At the same time, social determinants including HIV stigma and other intersecting stigmas and social inequalities are increasingly seen as fundamental barriers to accessing and engaging in HIV treatment and prevention. As succinctly stated by Dr. Anthony S. Fauci, Director of NIAID, in a recent interview, “stigma is the enemy of public health” [17]. This recognition has been matched by renewed calls for improved approaches to measuring social determinants and the multifactorial pathways through which they operate over time [18].

## Methods

In May of 2019, NIMH, in collaboration with American University’s (AU) Center on Health, Risk and Society and the DC Center for AIDS Research, sponsored a symposium to bring together the six study teams funded through the above referenced RFA on the social determinants of HIV to share accomplishments, distill lessons learned and reflect on the state of the science with other research experts, community members and government officials. Approximately 100 individuals from 39 distinct governmental, nonprofit and academic agencies attended the symposium.

The event began with remarks from AU President Sylvia Burwell, former Secretary of the United States Department of Health and Human Services (DHHS) and Dr. Dianne Rausch, Director of NIMH’s Division of AIDS Research. Both stressed the urgency and opportunity of the current moment. President Burwell highlighted an initiative from her time at DHHS, “Public Health 3.0”, which acknowledged and addressed “how a patient’s health interacts with economic well-being, education, housing, or the safety of their neighborhood.” Dr. Rausch outlined components of the current US National HIV/AIDS Strategy for the United States: Updated to 2020. The updated Strategy seeks to move us closer to a time when new HIV diagnoses or transmissions are rare and when they do occur, every person regardless of social or economic condition will have equal access to treatment and care that is free from stigma and discrimination.

These opening remarks were followed by presentations from each of the six investigative teams. Presentations focused on causal inference, multi-level analysis and mathematical modeling (Models); geospatial analytics and ecological momentary assessments (Maps); and in-depth exploration and measurement of social and structural determinants including inequalities and stigmas (Measures). Each session was hosted by an expert discussant who framed presentations and engaged meeting participants in a targeted follow up conversation at the conclusion of the presentations. A final session hosted by Dr. Paul Gaist, Senior Advisor to the Director within NIH’s Office of AIDS Research, was devoted to synthesis of the day’s discussion. The goal of this session, which included academic, government and community representatives, was to identify cross-cutting themes related to the social determinants of HIV, and opportunities for continued methodologic advancement.

### Models

Prediction models bring epidemiological, statistical and demographic estimation methods together to provide a more integrated analytical framework to examine how social determinants impact the epidemic, or how they produce different scenarios across countries or regions. Dr. Haidong Wang (University of Washington) and his team are linking social determinants of HIV with key transmission and demographic factors to provide a more precise burden estimation in global and national prediction models and ultimately to discern the contribution of social determinants as drivers of HIV. Dr. Wang and colleagues are developing models that include measurements of important components such as income or education when estimating HIV incidence, prevalence or mortality to better gauge and monitor their impact. Modeling could be used to test the impact of different levels of social determinants, along with other key parameters to understand the social dynamics of HIV transmission or morbidity and mortality, and how potential public policies could aid in minimizing or ending the HIV epidemic.

Statistical models leverage single data sources to understand causal impact. For example, Drs. Audrey Pettifor and Marie Stoner (University of North Carolina, Chapel Hill) are leveraging data from a randomized controlled trial with over 2300 adolescent girls in rural South Africa (HPTN 068) to understand how social determinants such as access to education can increase or decrease risks for HIV acquisition. Through this mediation work, the team determined that much of the protective effect of school attendance in this population was explained by sexual networks (e.g. older partners) and how intervening on this exposure-outcome relationship can reduce the risk for HIV in a given population. Such advanced statistical techniques will help to further estimate the potential impact of different forms of social and structural interventions at various levels of scope and scale across differing geographic contexts. Lead discussant, Dr. Joseph Hogan (Brown University) pointed out the difference between prediction models like Dr. Wang’s work, which rely on “macro” data versus causal models such as Drs. Pettifor and Stoner’s research that rely on “micro” data to understand the relative importance of social determinants in global HIV disease burden estimates.

### Maps

Leveraging technology to understand how physical and social environments affect individuals and impact HIV outcomes was the focus of the second panel. Dr. Dustin Duncan (Columbia University) is using real-time geospatial methods to identify Global Positioning System (GPS)-defined activity space neighborhoods to better capture the breadth of marginalized men’s actual exposures to neighborhood-level risks. Typically, residential neighborhoods are used as a measure of environmental impact; however, participants often travel to other neighborhoods to maintain their various social (and sexual) networks. Such spatial mobility is particularly true for younger individuals. In this study, participants wear a GPS device following established protocols over time. Multiple GPS measures along with network-level measures are examined as predictors of HIV prevention outcomes, particularly among socially disadvantaged populations at substantial risk for HIV acquisition.

Utilizing Geographical Information System (GIS) data collection with Ecological Momentary Assessment (EMA) and integrating these findings with qualitative in-depth interviews is another promising approach by Drs. Benjamin Henwood and Eldin Dzubur (University of Southern California). Known as Geographically Explicit Ecological Momentary Assessment (GEMA), these researchers are utilizing this approach with transition-age youth (~ 18–25) who have experienced homelessness to test for the effects of environment and proximity to home on HIV risk behaviors. The team is filtering EMA responses to provide a personalized geospatial map rendering of EMA responses to discuss locations and contexts of risk among young adults now living in supportive housing arrangements. Such a mixed-methods approach emphasizes uniqueness-in-context and is useful in considering complex, interrelated influences that can exist on multiple levels. The team is investigating high and low risk environments within supportive housing to examine how individual and contextual factors interact to influence risk. The lead discussant, Dr. Eric Rice (University of Southern California) noted that understanding where (physical environment) and how (social dynamics) people are living their moment-to-moment lives will help to inform interventions. However, participants emphasized the need for multi-level versus individual-level approaches to inform structural interventions that address the structural drivers and environments in which individuals found themselves.

### Measures

The panel on measurement of latent social constructs including dynamics of social inequality and stigma, emphasized the importance of grounding the development of unidimensional measures of complex constructs in social theory and the central role of in-depth qualitative research. Drs. Deanna Kerrigan and Wendy Davis (George Washington University), Clare Barrington (University of North Carolina, Chapel Hill) and colleagues from Johns Hopkins University are conducting mixed-method psychometric research to create a new valid and reliable measure of sex work stigma to examine its intersection with HIV stigma in predicting HIV treatment outcomes among female sex workers living with HIV in both the Dominican Republic and Tanzania. The team is also using longitudinal qualitative in-depth interviews (integrating both thematic and narrative approaches) and structural equation modeling to assess pathways from social determinants to biological outcomes. The importance of longitudinal designs was underscored to validate measures, elucidate mechanisms and pathways between social determinants and HIV outcomes, and meaningfully capture change over time.

Drs. Kim Blankenship (American University) and Jonathan Purtle (Drexel University) are investigating the intersecting impacts of mass incarceration, housing stability and subsidized housing policies on HIV outcomes. The team is expanding policy measurement and analysis beyond simple dichotomous measures to quantitatively account for differences in exposure to constellations of “micro policy provisions” that might increase health risk. Development steps include identifying cities, housing authorities and housing policies (“on the books”) for coding according to their degree of restrictiveness. Local housing authority policies with disparate impacts that restrict access to public housing among people with criminal justice histories may increase HIV risk and lead to negative health outcomes. The team will also use findings from longitudinal mixed-methods approaches with multiple stakeholders including policy makers and community members, to clarify how those who interpret, implement and are impacted by such policies, actually understand them. Dr. Susan Sherman (Johns Hopkins University) and audience members highlighted the importance of macro-level determinants such as economics and laws or legal frameworks in such longitudinal analyses.

## Discussion

An interdisciplinary panel representing research (Dr. Lisa Bowleg, George Washington University; Dr. Yeycy Donastorg, Instituto Dermatológico Dominicano y Cirugía de Piel “Dr. Humberto Bogaert Díaz”), community (Ms. Martha Sichone Cameron, The Women’s Collective) and government perspectives (Dr. Paul Gaist) was moderated by Dr. Gaist. Cross-cutting and higher-level themes were discussed and largely focused around the following areas.

### Social Theory

As the Social Determinants of Health framework gained attention in the early 2000s, so too did the call from social scientists for greater reflection regarding the theoretical underpinnings of this conceptual framework. Scholars from a variety of disciplines and perspectives document the need to integrate social theory to more rigorously explore the social determinants of health. Specifically, there is a need for theory that addresses the dynamics and structures of power that are at play in relation to socio-economic position, race/ethnicity, gender, sexual orientation, living and working conditions and other social determinants. When researching the social determinants of health, the focus should be on the physical and social environment that shapes the probability of a given health outcome for groups rather than on intra-personal decision-making processes and behaviors. Leveraging technology to explore where and how places and spaces influence HIV outcomes is a good example. Equally important is the need to move beyond the socioecological model that simply lists a variety of social determinants to include the mechanisms and pathways by which these social factors impact interpersonal processes and individual outcomes. Predictive and causal modeling studies could aid in such elucidation.

Potvin and colleagues [19] argue that there is an equally great need to integrate social theory into formative research focused on program development with evaluation research focused on examining the effectiveness of public health policies and programs that address the social determinants of health. As an example, the Justice, Housing and Health Study (JustHouHS) study taking place in New Haven, Connecticut (USA) and represented in this supplement [20] as explicitly drawn on social theory to guide its conceptualization and implementation. The study is investigating how intersecting social determinants, including mass incarceration, housing instability and housing policies, are related to the overall health and HIV-related sexual risk of people recently involved in the criminal justice system and/or seeking or living in subsidized housing. The study uses a mix of surveys and in-depth interviews conducted, every 6 months, over a period of two years. To better understand power dynamics and structural constraints, the project also conducts qualitative research with policy makers working in these different sectors and archival analysis of policy documents related to both housing and incarceration.

### Community Engagement

To effectively address social determinants of HIV, community engagement is essential. Community engagement is the process of working collaboratively with a given geographic, social or symbolic community. As Valdisseri and Holtgrave [21] note, “Merely acknowledging that social and economic determinants influence health is not sufficient. To truly understand the breadth and relative importance of contextual issues, we must seek-out and attend to the first-hand knowledge of those individuals and communities who we are trying to reach with these interventions.” Community partners are uniquely equipped to identify the relevant social and economic factors that challenge their health and illuminate the intricate ways in which mapping studies could explore how these factors intersect and impact HIV outcomes over time. Engaging communities most impacted by the social determinants of HIV can guide how research questions in modeling studies are framed to address them to ensure that they focus on the highest public health priorities. Having communities weigh in on scientific design and proposed research methodologies also ensures that studies are feasible and results fully representative. A meaningful partnership, built on longstanding collaborations between investigators and the communities they aim to serve, must underlie research on the social determinants of HIV at every stage, from determining methods and approaches to conducting research to analyzing and interpreting results.

An example of community engagement related to the social determinants of HIV in this supplement [22] is a study seeking to examine social dynamics over time among female sex workers living with HIV in the Dominican Republic and Tanzania. One of the aims of the study is to develop a valid and reliable scale to assess sex work stigma that can be used across settings. The study team first utilized in-depth interviews to understand the context, dimensions and dynamics of sex work stigma within female sex workers’ daily lives in each country. Once specific domains of sex work stigma were identified by community members, those experiences helped to generate potential scale items, and cognitive debriefing interviews were then used to ensure that survey questions and items associated with these domains were expressed in a manner that was understandable and acceptable to the community.

This step in the process also led interview participants, particularly in the Dominican Republic where community-driven responses to HIV among female sex workers have a long history, to emphasize the importance of not only internalized, anticipated or enacted stigma related to sex work, but also positive aspects including the ways in which women find dignity in their work as they support themselves and their families. As a result of these interviews and factor analysis, an additional scale domain of resisted sex work stigma or “dignity” was developed. Structured survey assessments were then used to quantify the level of these four sex work stigma domains, and to examine their relationship with related constructs such as HIV stigma, depression and anxiety, gender-based violence, as well as social cohesion. Item Response Theory (IRT) was then used to create context-specific IRT-adjusted domain scores to ensure that the measure is responsive to the unique needs and diversity across sex worker communities.

### Mixed Methods

Mixed methods research, or the use and integration of multiples forms of qualitative and/or quantitative data, is increasingly seen as an essential approach to address the complexities of responding to HIV, particularly to understand and address the social determinants of HIV [23]. Underlying the growth of mixed methods research is an enhanced understanding of the importance of having multiple types of data to contextualize and/or explain what we observe in everyday life including how various forms of social inequality, exclusion and/or stigmatization influence HIV risk and outcomes within or across a given population group(s) or setting(s). Multiple types of data (e.g. close vs open-ended) help to make visible the ways in which power discrepancies may impede health promoting behaviors for groups of people who experience systematic disadvantage or discrimination. Integrating in-depth interviews with survey assessments, for example, has the potential to make visible the daily struggles of marginalized groups to the extent that there is mindfulness and intentionality among researchers to effectively partner with communities to select and implement methodologic innovations.

An example from this supplement [24] of innovative mixed methods research on the social determinants of HIV includes the Log My Life study, which is examining how young adults who have experienced homelessness are exposed to environments that contribute to HIV risk behavior in Los Angeles, California. The study uses geographically explicit EMA through cell phone technology to document physical location, social context and HIV-related risk behavior of participants over time through daily (up to 8/day) surveys and diaries. In addition, a subsample of participants, specifically those who indicate that they are engaging in risky behaviors, complete an in-depth qualitative interview using an interactive, personalized geospatial map rendering of EMA responses to further understand the nuanced nature and dynamics of risk. Integrating quantitative and qualitative data collection in the study allows for a more complete understanding of differences in the HIV risk environments between homeless and housed young adults. The novel approach also helps prevent recall bias and enhance ecological validity given the moment to moment recording of physical spaces and social interactions.

## Conclusion

Throughout its history, social inequalities have defined the landscape of the HIV global pandemic. Structural drivers such as intersecting stigmas, poverty, and gender inequality are woven throughout the epidemic’s course, securing again and again the disparate impact on the vulnerable and marginalized. This pattern is seen in the expression of other pandemics, such as COVID-19, and in a host of infectious and chronic diseases [25, 26]. The HIV epidemic and research aimed at combatting it have been ground-breaking and unprecedented. At this juncture, the fight to contain HIV through understanding and intervening against its social and economic determinants, offers a unique opportunity both for the field of HIV as well as for the fight to curb other infectious and chronic disease epidemics where social determinants play an outsized role.

The research papers from the study teams, and the cross-cutting quantitative and modeling papers underscore how the social determinants of HIV, and the ways in which they may intersect, are complex but not chaotic. There are discernible patterns and dynamics that innovative methodologies and meaningful partnerships between researchers, government and communities can be measured, understood and intervened upon. Doing so is essential to bending the arc of the global HIV epidemic.

## References

[1] WHO. Rio Political Declaration at the World Conference on Social Determinants of Health; pp. 19–21 https://www.who.int/sdhconference/declaration/Rio_political_declaration.pdf?ua=1. Accessed 16 June 2019.

[2] Pellowski JA, Kalichman SC, Matthews KA, Adler N (2013). A pandemic of the poor: social disadvantage and the U.S. HIV epidemic. Am Psychol..

[3] Office of National AIDS Policy. National HIV/AIDS Strategy for the United States. Updated for 2020. Available from https://www.hiv.gov/sites/default/files/nhas-2020-action-plan.pdf. Accessed 1 June 2020.

[4] Ending the HIV Epidemic: A Plan for America. https://www.hiv.gov/federal-response/ending-the-hiv-epidemic/overview. Accessed 16 June 2019.

[5] UNAIDS. On the fast track to end AIDS. UNAIDS 2016–2021 Strategy. https://www.unaids.org/sites/default/files/media_asset/20151027_UNAIDS_PCB37_15_18_EN_rev1.pdf. Accessed 16 June 2019.

[6] NIH. Methodologies to enhance understanding of HIV-Associated social determinants (R01). https://grants.nih.gov/grants/guide/rfa-files/RFA-MH-16-200.html. Accessed 27 Nov 2020.

[7] NIH. Methodologies to Enhance Understanding of HIV-Associated Social Determinants (R21). https://grants.nih.gov/grants/guide/rfa-files/RFA-MH-16-205.html. Accessed 27 Nov 2020.

[8] UNAIDS. Global HIV and AIDS statistics — 2018 fact sheet. https://www.unaids.org/en/resources/fact-sheet. Accessed 16 June 2019

[9] Geter A, Sutton MY, Hubbard MD (2018). Social and structural determinants of HIV treatment and care among black women living with HIV infection: a systematic review: 2005–2016. AIDS Care.

[10] Feller DJ, Agins BD (2017). Understanding determinants of racial and ethnic disparities in viral load suppression. J Int Assoc Provid AIDS Care.

[11] Macdonald V, Verster A, Baggaley R (2017). A call for differentiated approaches to delivering HIV services to key populations. J Int AIDS Soc..

[12] Heestermans T, Browne JL, Aitken SC, VervoortFellerKlipstein-Grobusch SCK (2016). Determinants of adherence to antiretroviral therapy among HIV-positive adults in sub-Saharan Africa: a systematic review. BMJ Glob Health.

[13] Abgrall S, Amo J (2016). Effect of sociodemographic factors on survival of people living with HIV. Curr Opin HIV AIDS.

[14] UNAIDS. HIV prevention among key populations. https://www.unaids.org/en/resources/presscentre/featurestories/2016/november/20161121_keypops. Accessed 17 June 2019.

[15] UNAIDS. Miles to go. Closing gaps, breaking barriers, righting injustices. Global AIDS update 2018. UNAIDS, https://www.unaids.org/sites/default/files/media_asset/miles-to-go_en.pdf. Accessed 16 Jun 2019.

[16] Huang YA, Zhu W, Smith DK, Harris N, Hoover KW (2016). HIV pre exposure prophylaxis, by race and ethnicity- United States, 2014–2016. MMWR Morb Mortal Wkly Rep..

[17] CSIS. AIDS 2020. Anthony Fauci: transcending administrations. https://soundcloud.com/csis-57169780/anthony-fauci-transcending-administrations. Accessed 13 June 2019.

[18] Palmer RC, Ismond D, Rodriquez EJ, Kaufaman JS (2019). Social determinants of health: future directions for health disparities research. Am J Public Health.

[19] Potvin L, Gendron S, Bilodeau A, Chabot P (2005). Integrating social theory into public health practice. Am J Public Health.

[20] Blankenship K, Rosenberg A, Keene DE, Dawson AJ, Groves AK, Schlesinger P. Women’s relationships and HIV in the social context of mass incarceration and housing vulnerability. AIDS Behav**.** 10.1007/s10461-021-03238-4 PMC8484381 33796957

[21] Valdiserri RO, Holtgrave DR (2019). Ending HIV in America: not without the power of community. AIDS Behav..

[22] Kerrigan D, Karver TS, Barrington C, Davis W, Donastorg Y, Perez M, Gomez H, Mbwambo J, Likindikoki S, Shembilu C, Mantsios A, Beckham SW, Galai N, Chan KS. Development of the experiences of sex work stigma scale using item response theory: implications for research on the social determinants of HIV. AIDS Behav**.** 10.1007/s10461-021-03211-1 PMC8446100 33730252

[23] Auerbach JD, Parkhurst JO, Cáceres CF (2011). Addressing social drivers of HIV/AIDS for the long-term response: conceptual and methodological considerations. Glob Public Health.

[24] Madden DR, Semborski S, Dzubur E, Redline B, Rhoades H, Henwood H. Examining HIV risk and exchange sex among current and formerly homeless young adults. AIDS Behav. 10.1007/s10461-021-03364-z PMC8542003 34302283

[25] Abrams EM, Szefler SJ (2020). COVID-19 and the impact of social determinants of health. Lancet Respir Med..

[26] Cockerham WC, Hamby BW, Oates GR (2017). The social determinants of chronic disease. Am J Prev Med..

